# Experimental, RSM modelling, and DFT simulation of CO_2_ adsorption on Modified activated carbon with LiOH

**DOI:** 10.1038/s41598-024-64503-9

**Published:** 2024-06-12

**Authors:** Marziyeh Ahmadi, Fatemeh Bahmanzadegan, Mohammad Qasemnazhand, Ahad Ghaemi, Hamid Ramezanipour Penchah

**Affiliations:** School of Chemical, Petroleum and Gas Engineering, Iran University of Science and Technology, Tehran, Iran

**Keywords:** CO_2_, Activated carbon, Lithium hydroxide, RSM, DFT, Environmental chemistry, Environmental impact, Chemical engineering

## Abstract

This research investigates the enhancement of CO_2_ adsorption capacity through the use of modified activated carbon (AC) with LiOH, focusing on operational conditions and adsorbent properties. Response Surface Methodology (RSM) is employed to optimize process parameters for maximizing CO_2_ adsorption capacity. The study considers temperature, pressure, LiOH concentration for modification, and adsorbent weight as independent variables across five levels. Analysis of Variance reveals that LiOH concentration, adsorbent quantity, pressure, and temperature significantly influence CO_2_ adsorption. Optimal values for temperature (30°C), pressure (9 bar), LiOH concentration (0.5 mol/L), and adsorbent weight (0.5 g) result in a maximal CO_2_ adsorption capacity of 154.90 mg/g. Equilibrium adsorption capacity is utilized for modeling, with the Freundlich model proving suitable for CO_2_ adsorption on LiOH-AC. Kinetic modeling indicates the second-order model's suitability for temperatures of 30 °C and 50 °C, while the Elovich model fits temperatures of 70 °C and 90 °C. Thermodynamic modeling at the optimized conditions (303 K and 6 bar) yields ∆H, ∆S, and ∆G values of adsorption as 12.258 kJ/mol, − 0.017 kJ/mol·K, and − 7.031 kJ/mol, respectively. Furthermore, structural considerations of AC are discussed alongside modeling and simulation, presenting the adsorption rate of CO_2_ and the binding energy index based on Density Functional Theory (DFT).

## Introduction

Carbon dioxide (CO_2_) emissions and world warming by greenhouse gases have been the biggest problems in the last decade. Over the years, studies have been carried out to capture, remove, and store CO_2_. The main methods of CO_2_ capture and removal are absorption^1^, adsorption^2^, membrane separation^3^, and cryogenic^4^. Membranes are less efficient and more complex to scale up for CO_2_ separation^5^. Cryogenic is used to separate CO_2_ gas; this method is most likely rejected for CO_2_ due to the high energy demand involved. Absorption with chemical solutions is the most mature technology for large-scale CO_2_ capture^6,7^. However, there are some issues related to this approach, such as equipment devaluation and high energy consumption^8,9^. Adsorption is an attractive method to overcome some disadvantages of the mentioned technologies^10^. Generally, the flexibility and efficiency of the adsorption process made it a suitable choice for gas purification. CO_2_ adsorption is a viable method due to its high regenerative potential and low energy requirements^11^. Some materials used in CO_2_ adsorption such as carbon-based material^12–14^, silica^15,16^, zeolites^17^, Metal Organic Frameworks (MOFs)^18^, and metal oxides^19^. Among these materials, carbon-based porous materials such as activated carbon (AC) are widely used to CO_2_ adsorption^20–22^. AC is a highly microporous material with a large surface area for removing pollutants. Research showed that CO_2_ adsorption by modified AC with active substances is higher than pure AC at room temperature^23^. Alkaline bases such as sodium, calcium, and potassium hydroxides are exciting due to their fast reaction with CO_2_ molecules to increase adsorption^24,25^. Modification of AC with them, improved surface area. Potassium hydroxide is one of the active substances introduced in modifying AC to adsorb more CO_2_^26^. Karbalaei et al. presented NaOH-AC in a batch reactor. Experiments are done at 2–10 bars, 20–80°C, and the concentration of NaOH solution is 10–40 percent. The best CO_2_ adsorption capacity is achieved at 104.32 mg/g^27^. Casco et al. modified AC with KOH. CO_2_ adsorption capacity at 298 K and pressure of 45 bar was obtained 1500 mg/g^28^. Tan et al. investigated the CO_2_ adsorption capacity of NaOH-AC at 35 °C, which was found to be 27.10 mg/g^29^. Dehkordi et al. investigated the effect of NaOH-AC on CO_2_ adsorption. The results showed that the optimum condition was 51.41 mg/g, which indicates a more than 142% increase in CO_2_ adsorption capacity^30^. Tovar et al. conducted experiments on LiOH supported on high-surface-area carbons for CO_2_ adsorption in water-saturated environments. The highest achievable loading of LiOH on AC was 30 wt%, and the CO_2_ capacity under water-saturated conditions was 3.4 mol/kg^31^. Krishnan et al. used LiOH and Ca (OH)_2_ to modify AC for CO_2_ adsorption^32^. Table 1 indicates the comparison of CO_2_ adsorption capacity of modified activated carbon.

**Table 1:** Review of studies on different modified AC for CO_2_ adsorption.

Material	BET surface area, m^2^/g	P (bar)	T (k)	Adsorption capacity (mg/g)	Ref
Black locust-KOH	–	1	273	316.431	^40^
Sugarcane bagasse-LiOH	–	1	273	242.055	^41^
AC/Zeolite 13X	656.0	0.05	303	233.693	^42^
Pd-AC/MOF-74(Co)	1088.0	32	298	502.594	^43^
AC/CueZn	599.4	1	303	99.462	^44^
AC nanofibers/MgO	413.0	1	298	91.541	^45^
AC/CuO	1954.0	1	303	298.388	^46^
AC/NiO	1954.0	1	303	285.185	^46^
AC/hydrotalcite	441.0	3	473	50.171	^47^
AC/LiOH	781.8	9	303	154.960	This study

In the present study, activated carbon (AC) was modified with lithium hydroxide (LiOH) to improve its CO_2_ adsorption capacity. First, the impregnation of AC with LiOH was modeled to explore its effect^33,34^. Then, the binding energy between CO_2_ and the designed structure was calculated based on density functional theory (DFT) simulations^35^. By calculating the IR spectrum for the modeled structures, the stability of the structures was assessed^36^. Response Surface Methodology (RSM) was employed to determine the optimal conditions for the highest CO_2_ adsorption capacity and to design and analyze the experimental results of the variables for AC and LiOH-AC^2,37^. Additionally, kinetic, isotherm and thermodynamic modeling were carried out using the experimental equilibrium of pressure and adsorption capacity. It is important to note that RSM has not been previously used for optimizing modified activated carbon with varying percentages of LiOH concentration. Furthermore, DFT in existing literature has not been applied to CO_2_ capture for this specific system; instead, RSM has typically been used for activated carbon applications related to dye removal^38,39^. This study fills these gaps by applying RSM to optimize LiOH-AC for CO_2_ capture and utilizing DFT to understand the interactions in this novel system.

## Materials and methods

### Materials

LiOH Powder was purchased from Merck Chemical Co., and purified carbon dioxide gas (99.98%) was supplied by Sabalan Gas Co. (Tehran, Iran). Granular AC with a particle size of 0.5–2.2 mm is purchased from the Iranian market.

### Adsorbent preparation

AC was modified commercially by LiOH saturated solution. Typically, 10 g of AC was dissolved in 100 mL of a solution with different concentrations of LiOH (0.5–2 mol/L) for 2 h at room temperature. After that, the mixture was placed at room temperature for 22 h and then, it was filtered and dried at 85 °C for 6 h. The prepared samples were identified based on the concentration of LiOH used in the modification process, corresponding to 12, 24, 36, and 48 L, indicating the percentage of LiOH used.

### Characterization

The surface and porosity of AC and 24Li-AC samples are measured using the Brunauer–Emmett–Teller (BET) method. This is done by analyzing nitrogen adsorption/desorption isotherms at 77 K with a BELSORP-mini II analyzer. FTIR analysis was accomplished using Perkin Elmer, Model 2000 FTIR, USA, to identify the presence of functional groups for unmodified and LiOH-AC. The effect of LiOH modification on the morphology of AC was determined using FE-SEM analysis (FEI Sirion 200).

### Adsorption setup

**Figure 1:**
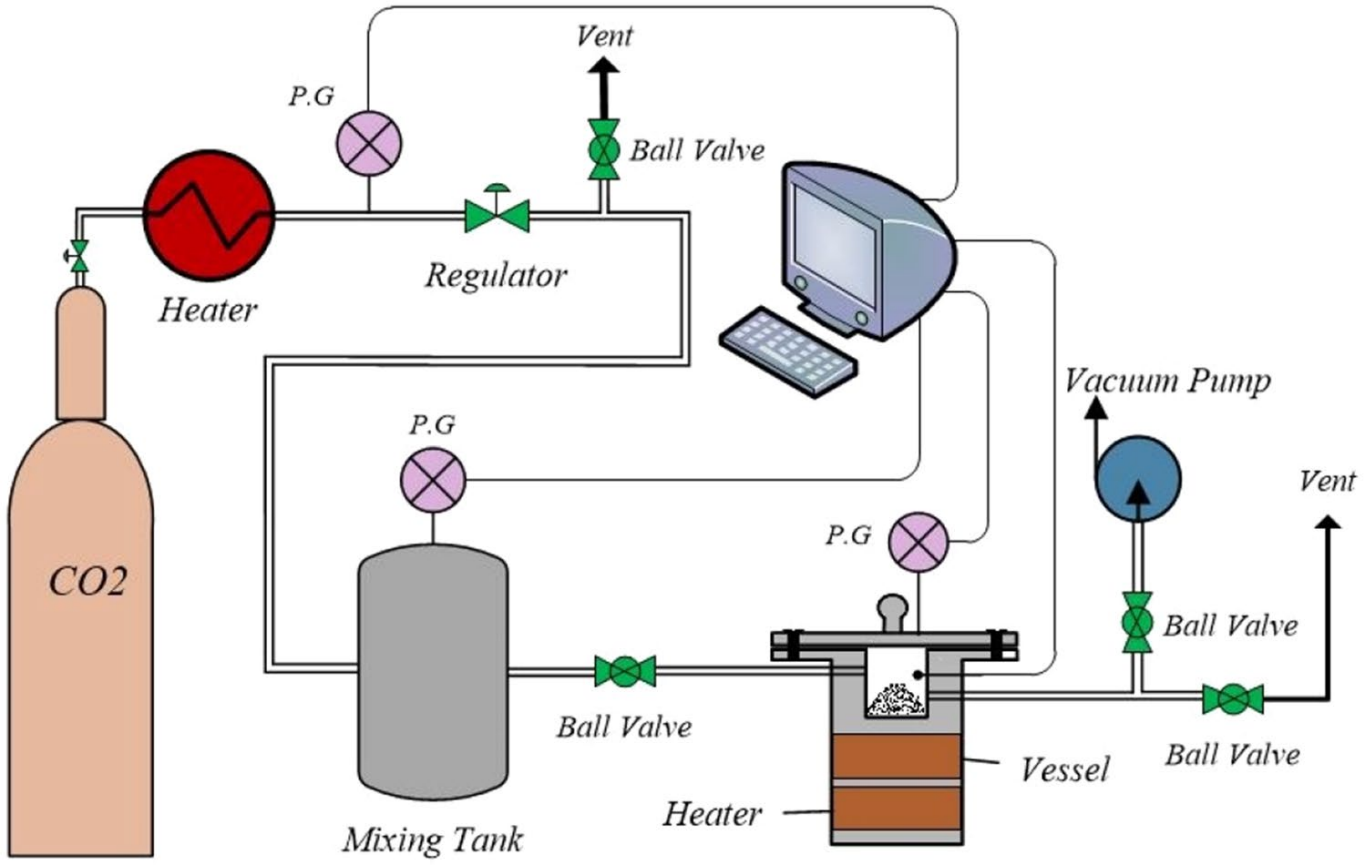
CO_2_ adsorption setup.

The experimental setup's schematic diagram is displayed in Fig. 1. At the start of a process, CO_2_ is transferred to a reactor chamber using pressure flow monitors. The reactor has a length of 9 cm, an inner radius of 3 cm, and an internal volume of 255 cm^3^. Temperature is controlled by a thermocouple. During the one hour process, which involves a solid adsorbent, the temperature and pressure changes in the reactor are analyzed and controlled. The reactor used in this process operates under specific conditions. These include a pressure range of 1 to 9 bar, a temperature range of 30 to 90°C, and a fixed amount of 1 gr of adsorbent. The CO_2_ is captured through the solid adsorbent material, which leads to a reduction in pressure. The rate of adsorption is calculated by measuring the difference between the initial and final CO_2_ pressure using a gas sensor. Finally, the adsorption capacity is measured using specific Eqs. (1) and (2).

1 $$q=\frac{{m}_{i}-{m}_{f}}{w}=\left(\frac{V{M}_{w}}{Rw}\right)\left(\frac{{P}_{i}}{{Z}_{i}{T}_{i}}-\frac{{P}_{f}}{{Z}_{f}{T}_{f}}\right)$$

2 $$Z=1+\frac{BP}{RT}$$

where m_*i*_ and m_*f*_ refer to the initial and final mass of adsorbed gas, respectively. V, R, w, M_w_, P, Z, T, and B are adsorption vessel volume, universal gas constant, mass of adsorbent, gas molecular weight, pressure, compressibility factor, temperature, and virial second coefficient, respectively.

### Response surface methodology (RSM)

RSM was applied to analyze the experimental adsorption data to decrease the cost and increase the quality of the experiment and process optimization^48^. In this study, four independent parameters (Table 2) are considered in the RSM method.

**Table 2:** Variables used in RSM.

Independent numerical Variables	Unit	Symbol	Variable levels
Temperature	°C	X_1_	30, 50, 70, 90
Pressure	bar	X_2_	1, 3, 6, 9
Adsorbent amount	gr	X_3_	0.5, 1, 1.5, 2
LiOH concentration	mol/L	X_4_	0.5, 1, 1.5, 2

RSM-CCD (Central Composite Design) produces an empirical equation based on independent parameters in the experiment range (Eq. (3)) that explain the response of the design.

3 $$y={\beta }_{0}+\sum {\beta }_{i}{X}_{i}+\sum {\beta }_{ii}{X}_{i}^{2}+\sum {\beta }_{ij}{X}_{i}{X}_{j}$$

where β_0_, β_i_, β_ii_, and β_ij_ are constants of the empirical equation.

### DFT simulation

The simulation with density functional theory (DFT) calculations was used to investigate the effect of impregnating active carbon with LiOH on CO_2_ adsorption. The stability of the structures has been checked by calculating the IR spectrum for the modeled structures^36^. The electron density modeling calculations of the simulated structures were performed using the LANL2DZ basis set and B3LYP function. Gaussian 98 software was used for DFT calculations.

## Result and discussion

### Characterization

**Figure 2:**
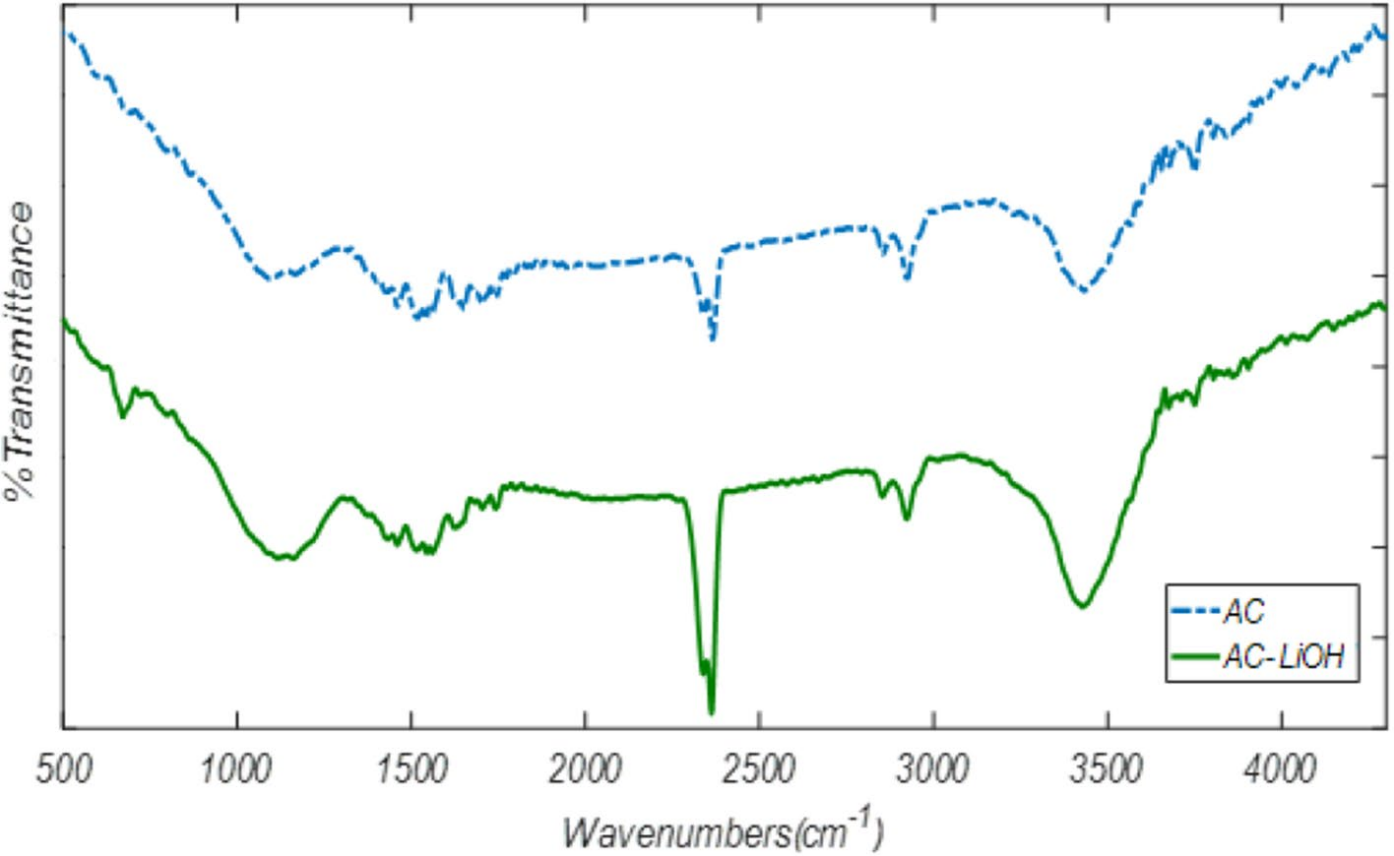
FTIR spectrum of unmodified and 24Li-AC.

The FTIR spectrum evaluates functional groups. Figure 2 shows the FTIR spectrum for AC before and after LiOH modification. According to the FTIR spectrum of 24Li-AC, 3426.73, 2361.96, 1560.42, and 1161.27 cm^−1^ peaks were related to C–OH, C-H, C≡C, and C=C bonds, respectively. The results show that the modification of the AC with LiOH has been well justified. According to the figure, it can be said that the peaks before and after active carbon modification in 2356 cm^−1^ have a difference, which is due to higher CO_2_ adsorption in the LiOH modified than unmodified AC.

**Figure 3:**
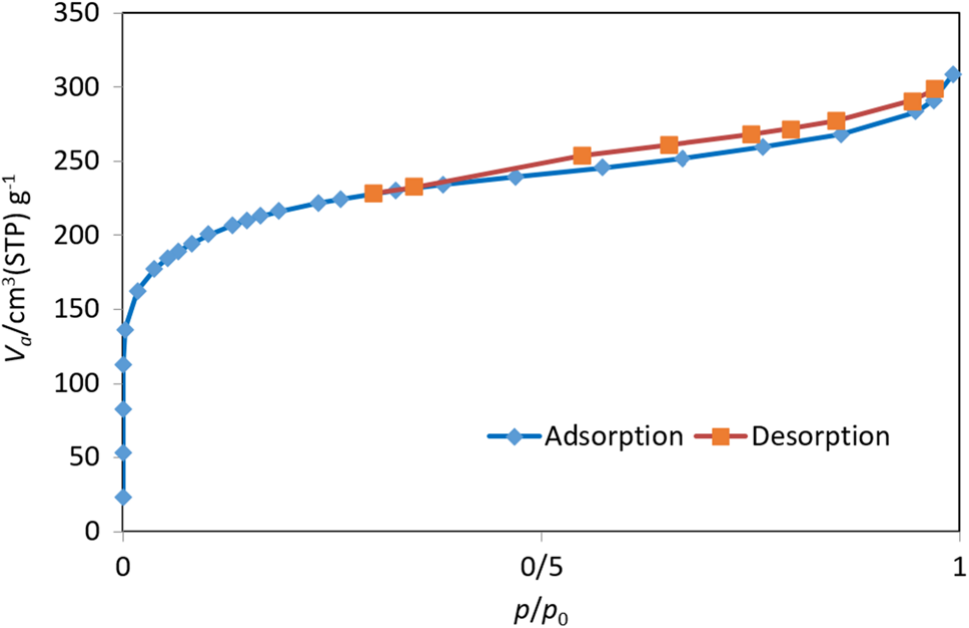
Adsorption of nitrogen at a temperature of 77 K on AC modified by LiOH.

Adsorbent structure properties such as surface area and pore volume were performed by BET analysis and shown in Fig. 3. The BET analysis of unmodified and LiOH-AC is listed in Table.

According to the results in Table 3, surface parameters increase after AC modification. The BET surface area of AC used in this investigation was determined to be 624.55 m2/g, while after modification, the BET surface area obtained 781.84 m^2^/g. The reason for the increase in the modified adsorbent level is the mesoporous properties of LiOH powder. The reaction of hydroxide lithium with AC increases the adsorbent level and improves the adsorbent level.

**Table 3:** Surface area and pore size adsorbent preparation.

Parameter	AC	24Li-AC
S_BET_ (m^2^/g)	624.55	781.84
V_t_ (cm^3^/g)	0.440	0.475
V_p_ (cm^3^ /g)	0.057	0.219
Pore size (nm)	1.250	2.432
Micropore area (m^2^/g)	37.72	175.98
The average pore diameter (nm)	0.658	1.290

**Figure 4:**
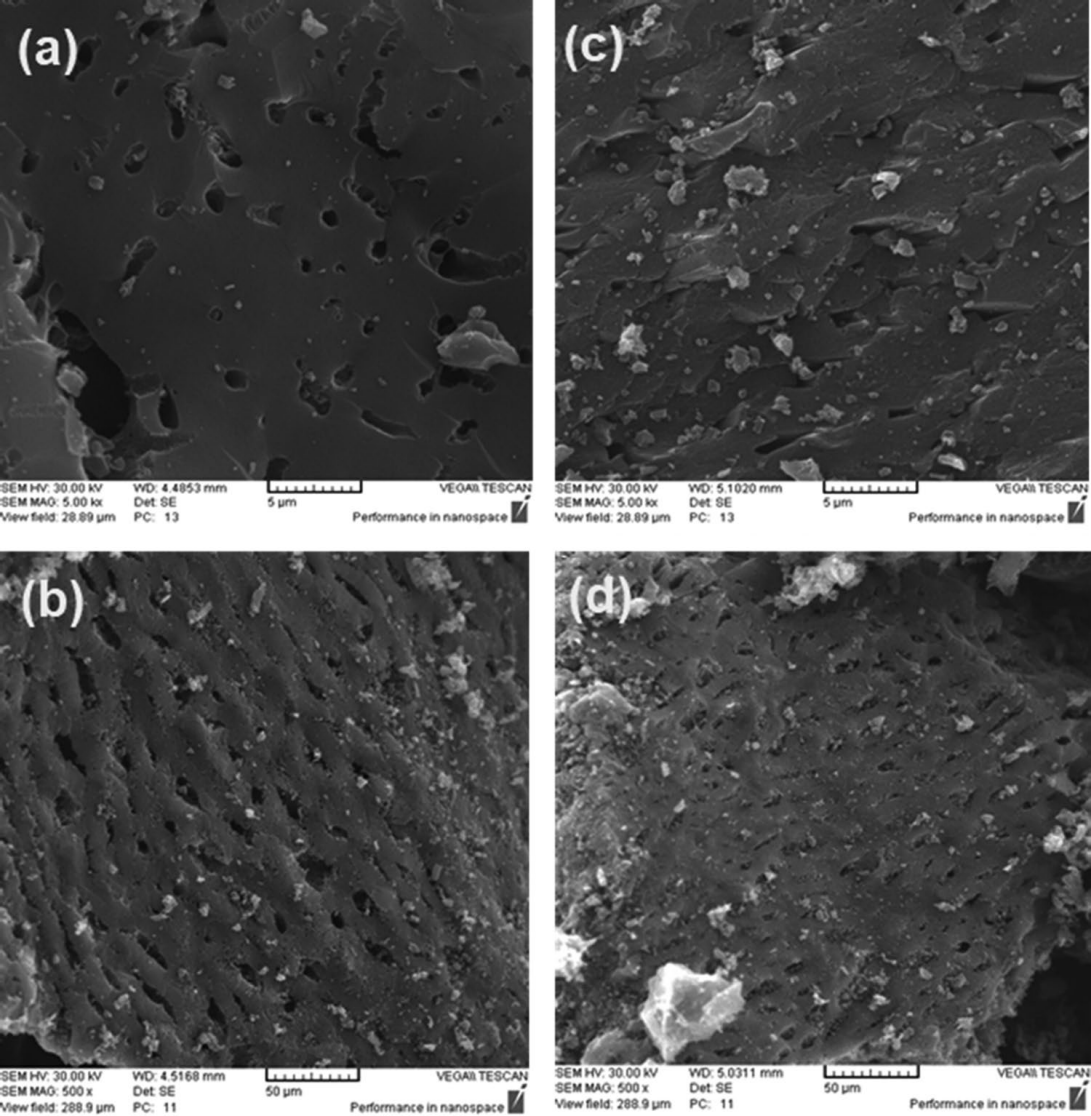
SEM analysis of AC: (**a**) AC in 5μm, (**b**) AC in 50 μm, (**c**) AC modified with LiOH in 5 μm, and (**d**) AC modified with LiOH in 50 μm.

Figure 4 shows the morphological structure of AC and modified AC with 24%LiOH (24Li-AC) at 50 and 500 µm magnifications. The activated carbon surface's porosity and the adsorbent surface's uniformity with different pore sizes are specified. According to these figures, it can be concluded that the adsorbent porosity in the unmodified case is less than the modified adsorbent, which indicates that LiOH penetrates inside the AC and causes an increase in the surface area and porosity. The 24Li-AC adsorbent has highly cracked surfaces with distinct pore sizes, indicating its suitability for CO_2_ adsorption.

### Analysis of variance (ANOVA)

ANOVA results of RSM predicted model versus temperature, pressure, LiOH concentration, and adsorbent weight are presented in Table 4. As shown, the predicted model P-Value < 0.0001 determines the model's significance. A, B, C, D, AB, AC, BC, BD, A^2^, and B^2^ are significant model terms in this model. The F-value of the model performed a comparison between the model analysis and the error. The obtained Model F-value of 1607.01 indicates that the model is significant.

**Table 4:** ANOVA results for the RSM model.

Source	Sum of Squares	df	Mean Square	F-value	p-value
Model	49,382.93	14	3527.35	1607.01	< 0.0001
A-T	21,678.29	1	21,678.29	9876.28	< 0.0001
B-P	14,770.27	1	14,770.27	6729.10	< 0.0001
C-LiOH	709.65	1	709.65	323.31	< 0.0001
D-Ads. weight	227.68	1	227.68	103.73	< 0.0001
AB	4053.09	1	4053.09	1846.52	< 0.0001
AC	206.28	1	206.28	93.98	< 0.0001
AD	0.9547	1	0.9547	0.4349	0.5196
BC	16.09	1	16.09	7.33	0.0162
BD	15.62	1	15.62	7.12	0.0176
CD	3.79	1	3.79	1.72	0.2089
A^2^	25.42	1	25.42	11.58	0.0039
B^2^	30.83	1	30.83	14.05	0.0019
C^2^	6.66	1	6.66	3.03	0.1021
D^2^	1.09	1	1.09	0.4987	0.4909
Residual	32.92	15	2.19		
Cor total	49,415.86	29			

The R^2^ value, which measures consistency between experimental and calculated data, is listed in Table 5. The predicted and adjusted R^2^ values are in reasonable agreement and close to 1. Adequate Precision value was obtained at 146.0413 (greater than 4) and is desirable^49^.

**Table 5:** Statistic values of CCD polynomial model for CO_2_ adsorption capacity.

Std. Dev	1.48	R^2^	0.9993
Mean	66.59	Adjusted R^2^	0.9987
C.V. %	2.22	Predicted R^2^	0.9973
		Adeq precision	146.0413

The empirical equation in terms of temperature (A), pressure (B), LiOH concentration (C), and adsorbent weight (D) is presented in Eq. (4).

4 $$q= 75.89822 + (-0.334119\times A)+ (19.12542\times B)+ (-23.27396\times C)+ (-6.30676\times D)+ (-0.141688\times A \times B)+ (0.172023\times A \times C)+ (0.011752\times A \times D)+ (-0.339938\times B \times C)+ (-0.339372\times B \times D)+ (-0.872207\times C \times D)+ (-0.003638\times A^2 )+ (-0.248435\times B^2 )+ (2.97372\times C^2 )+ (1.42341\times D^2 )$$

According to the results in Table 4, the calculated data were close to the experimental data quietly, and shows that obtained empirical and equation can explain and predict the CO_2_ adsorption process by unmodified and LiOH modified AC accurately.

### Parameters effect on CO_2_ adsorption capacity

**Figure 5:**
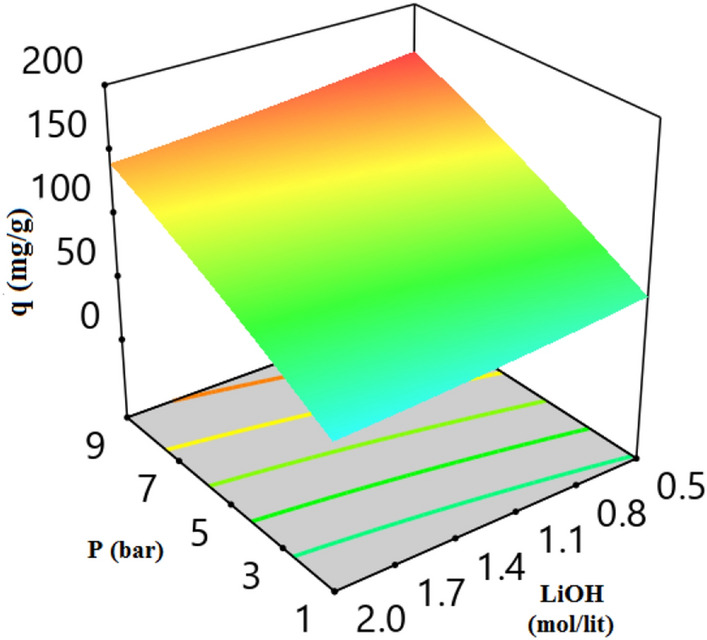
CO_2_ adsorption capacity with pressure and LiOH concentration at 30 °C and adsorbent weight of 0.5 gr.

**Figure 6:**
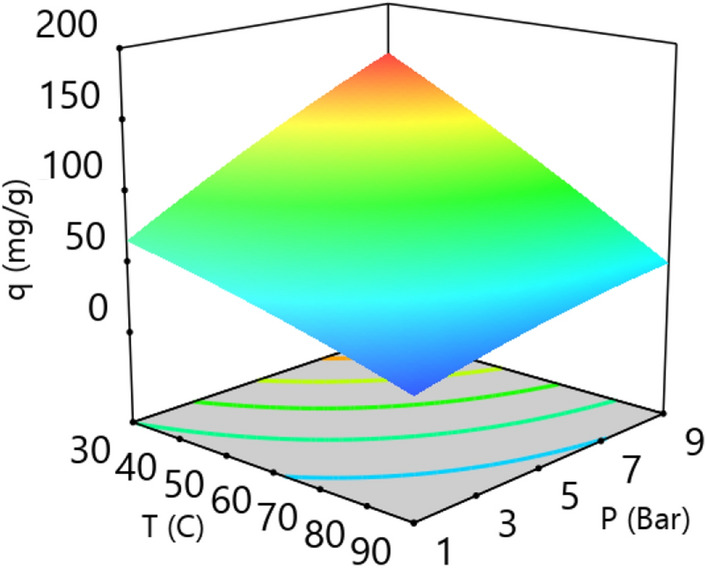
CO_2_ adsorption capacity with pressure and temperature at adsorbent weight of 0.5 gr and LiOH concentration of 0.5 mol/L.

**Figure 7:**
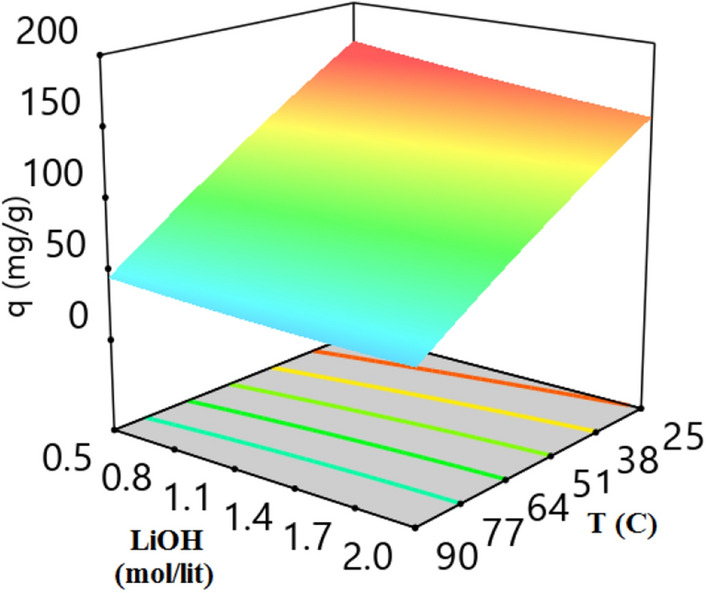
CO_2_ adsorption capacity with LiOH concentration and temperature at adsorbent weight of 0.5 g and pressure of 9 bar.

**Figure 8:**
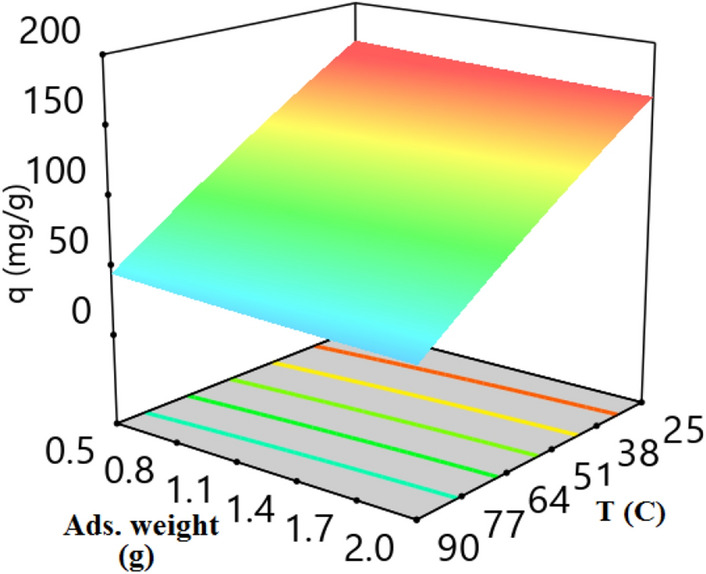
CO_2_ adsorption capacity with adsorbent weight and temperature at LiOH concentration of 0.5 mol/L and 9 bar.

**Figure 9:**
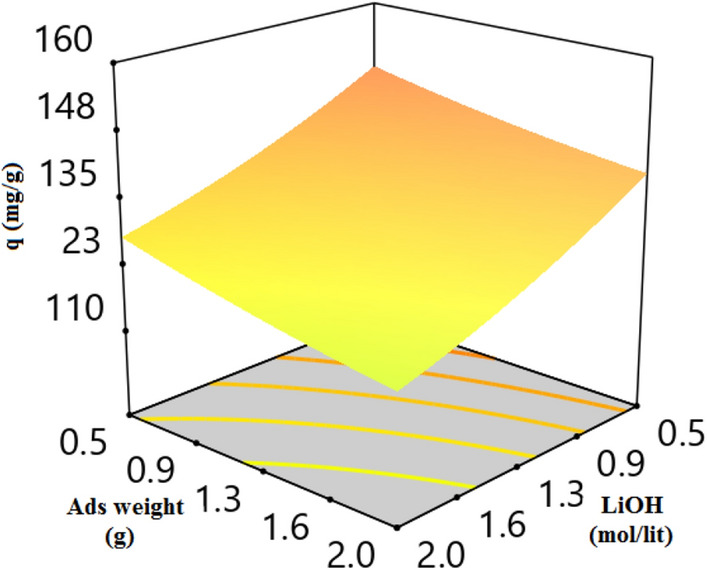
CO_2_ adsorption capacity versus adsorbent weight and LiOH concentration at 30 °C and 9 bar.

**Figure 10:**
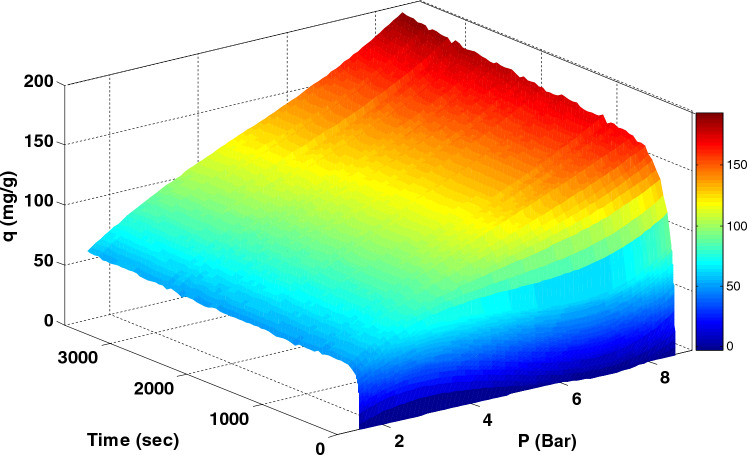
CO_2_ adsorption capacity at 30 °C versus time and pressure for 24Li-AC modified AC.

**Figure 11:**
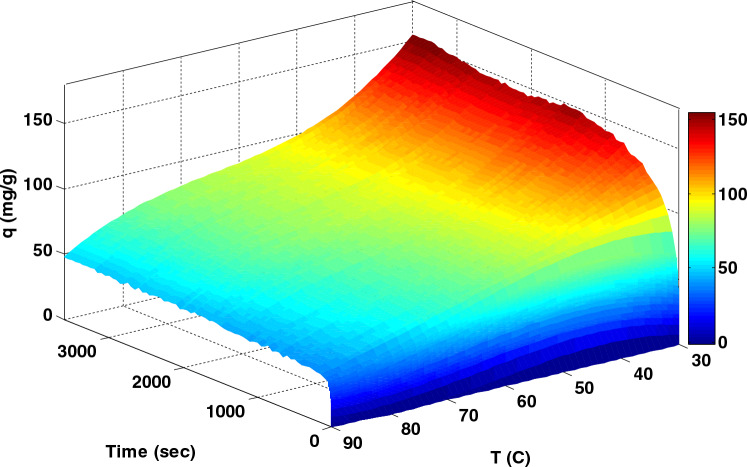
CO_2_ adsorption capacity at 6 bar versus time and temperature for 24Li-AC modified AC.

3-D plot of CO_2_ adsorption capacity and variables (temperature, pressure, LiOH concentration, and adsorbent weight) are presented in Figs. 7, 8, 9, 10 and 11. CO_2_ Adsorption capacity versus pressure, and LiOH concentration and temperature, and LiOH concentration are presented in Figs. 5 and 7 According to these figure, High concentrations of LiOH cause the adsorbent cavities to close and adsorption capacity to decrease. CO_2_ Adsorption capacity versus pressure and temperature is presented in Fig. 6, which shows that according to the nature of physical adsorption, the maximum amount of adsorption capacity take place at high pressure and low temperature and the minimum amount of that take place at low pressure and high temperature. According to Figs. 5 and 6, adsorption process occurs when the molecules in the gas or the liquid phase reach the solid surface and bond with adsorbent active sites. In gas bulk, the increase in pressure leads to an increase in the movement of the gas molecules to the adsorbent sites, and so, an increase in the velocity of equilibrium and adsorption reaction occurs. Therefore, pressure increasing cause to an increase in adsorption capacity (Fig. 7).

Figures 8 depicts the relationship between CO_2_ adsorption capacity, adsorbent weight, and temperature at a LiOH concentration of 0.5 mol/L and 9 bar. Similarly, Fig. 9 illustrates the relationship between CO_2_ adsorption capacity, adsorbent weight, and LiOH concentration at 30 °C and 9 bar. According to these figures, by increasing the amount of adsorbent, the amount of hydroxyl salts in the adsorbent increases, and the presence of these salts only leads to an increase in adsorbent weight without contributing to the progress of the carbon dioxide adsorption process.

### Optimization on adsorption process

In general, maximum CO_2_ adsorption capacity is necessary for adsorbent applications in the solid sorption process, and the optimized parameter points must be determined for each of adsorbents. Therefore, maximum CO_2_ adsorption capacity by LiOH modified AC, and the optimum values were obtained using desirability function value^50^. Consequently, the adsorption capacity (model response) was chosen as 'maximize', and independent parameters were selected 'within the range', to achieve the highest capacity. The maximum adsorption capacity was achieved at temperature of 30 °C, pressure of 9 bar, LiOH concentration of 0.5 mol/L with adsorbent weight of 0.5 g.

### Pressure effect on adsorption capacity

In order to evaluate the influence of adsorption time, 3-D plot of CO_2_ adsorption capacity versus pressure, temperature and time were plotted in Fig. 10 and 11. In Fig. 10, the highest CO_2_ adsorption capacity was obtained at a pressure of 9 bar, indicating that pressure positively affects the adsorption capacity. This trend is higher at higher pressure levels, so the equilibrium is not appreciably visible at higher pressures, and adsorption continues. The effect of pressure on improving the position of molecules in the empty places of the adsorbent and the unreacted adsorbent parts leads to an increase in the gas adsorption capacity. In general, with increasing pressure, the adsorption capacity also increases. Figure 11 shows the CO_2_ adsorption capacity at different temperatures: 30, 50, 70, and 90°C. The adsorption capacity increases with a decrease in temperature. It can be inferred that LiOH physically adsorbs CO_2_ at lower temperatures.

According to Fig. 10, the maximum adsorption capacity was detected at high pressure due to the entering of gas molecules in smaller pores with pressure increasing and low temperature with the physisorption mechanism.

### Temperature effect on adsorption capacity

**Figure 12:**
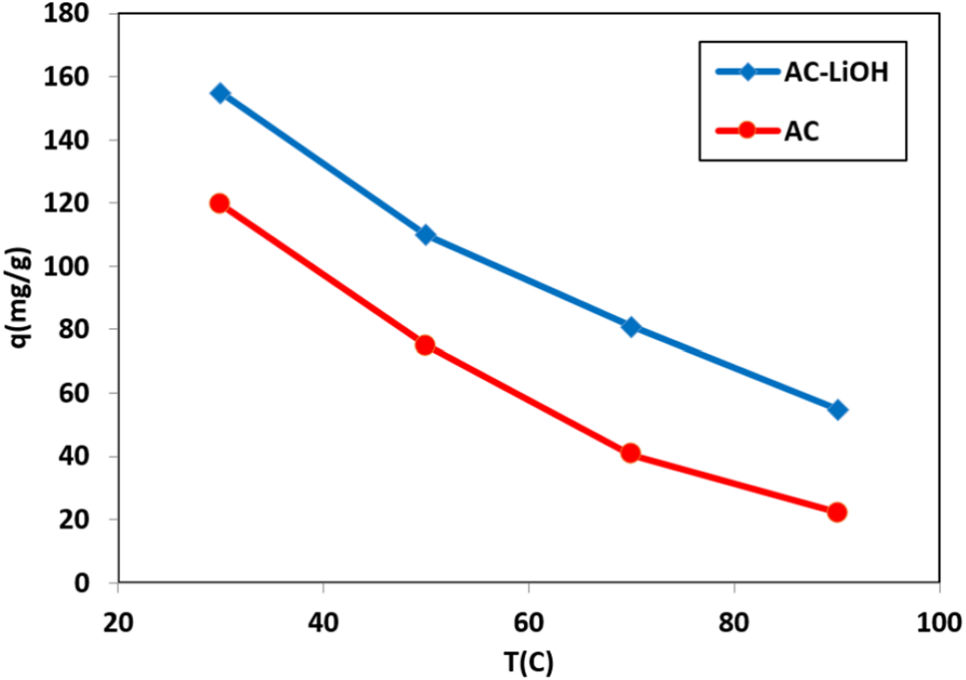
Comparison between unmodified and LiOH modified AC at different temperature and pressure of 6 bar.

**Figure 13:**
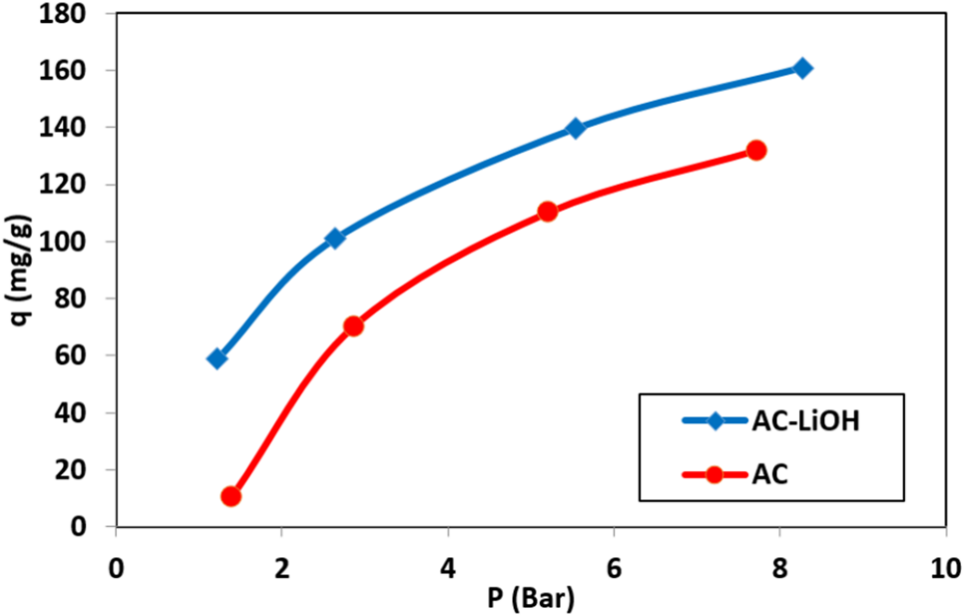
Comparison between unmodified and LiOH-AC at 30 °C and different pressure.

Comparison between AC before and after modification is presented in Figs. 12 and 13 at different temperature and pressure. According to these figures, the adsorbent modification due to the presence of LiOH has led to an increase in adsorption capacity by decreasing the temperature and increasing the pressure. This increase in adsorption has been constant at almost all temperatures and pressures, and has led to an increase of approximately 25% in the modification of the carbon dioxide adsorption capacity.

### Effect of LiOH concentration on adsorption capacity

**Figure 14:**
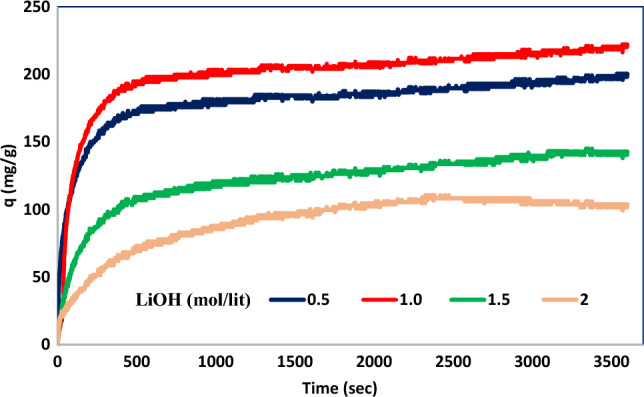
Effect of LiOH concentration percentage on CO_2_ adsorption capacity at 30°C and 6 bar.

The LiOH-ACs were utilized as adsorbents to investigate their performance for CO_2_ adsorption. The results are indicated in Table 6 and Fig. 14.

**Table 6:** Effect of LiOH concentration percentage on CO_2_ adsorption capacity at 30°C and 6 bar.

LiOH concentration (%)	LiOH concentration (molar)	Adsorption capacity (mg/g)
0	0.0	60.02
12	0.5	134.05
24	1.0	154.96
36	1.5	105.01
48	2.0	85.99

The results show that the highest adsorption capacity for CO_2_ is observed when using activated carbon modified by 24% LiOH (24Li-AC). The unmodified AC can adsorb 60.02 mg/g CO_2_. On the other hand, the 12%, 24%, 36%, and 48% LiOH-AC can adsorb 134.05, 154.96, 105.01, and 85.99 mg/g CO_2_, respectively.

It was found that modifying AC with one molar LiOH (or up to 24%) had a positive effect and significantly increased the capacity for CO_2_ adsorption. The experiment results indicated that 48Li-AC had a lower adsorption capacity. This could be attributed to the excessive concentration of LiOH solution that filled up the pores and cavities of the adsorbent. Additionally, the production of lithium salt hindered further carbon dioxide reactions. Since the 24Li-AC exhibited the best performance for CO_2_ adsorption, it was selected for other studies.

### Adsorption isotherm model correlation

It is crucial to identify the correct mechanisms and provide a quantitative description of thermodynamic equilibrium to optimize the design of the CO_2_ capture system. So, it's essential to understand the equilibrium process to predict how adsorption will occur accurately. Therefore, the experimental equilibrium data for carbon dioxide adsorbed in modified adsorbent was investigated using Langmuir, Freundlich and Dubinin–Radushkevich isotherm models. The isotherm model parameters are given in Table 7.

**Table 7:** Parameters of Isotherm Models for LiOH-AC.

Model	Equation	Parameter	Value	R^2^	AAER
Langmuir	${q}_{e}=\frac{({q}_{m}{k}_{L}{P}_{e})}{(1+{k}_{L}{P}_{e})}$	*q*_*m*_	469.03	0.9905	0.0362
		*k*_*l*_	0.101		
Freundlich	${q}_{e}={k}_{F}{{P}_{e}}^\frac{1}{n}$	*k*_*F*_	49.652	0.9956	0.0361
		*n*	1.433		
Dubinin –Radushkevich	${q}_{e}={q}_{m}{e}^{-\beta {\varepsilon }^{2}}$	*q*_*m*_	199.365	0.9573	0.1250
		*β*	39.659		
		*ε*	0.82		

**Figure 15:**
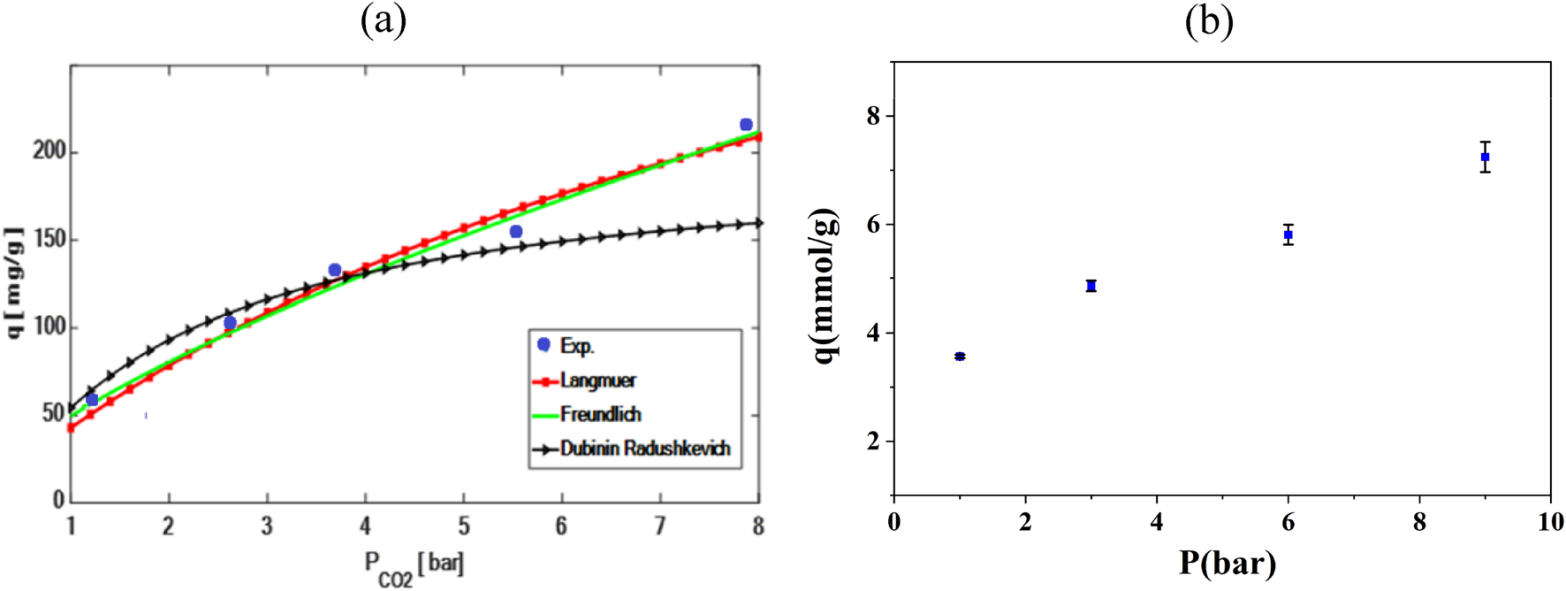
(**a**) Isotherm modeling of adsorption experimental data for 24Li-AC at 30 °C, (**b**) Error bar of experimental data.

CO_2_ adsorption isotherms at 303 K and pressures ranging from 1 to 9 bar are illustrated in Fig. 15a. The data demonstrate that higher pressures lead to increased CO_2_ adsorption rates. Table 6 summarizes the experimental results along with the R^2^ correlation coefficients for each isotherm model parameter. Utilizing nonlinear regression techniques and R^2^ values, the effectiveness of the theoretical isotherms in describing and predicting the adsorption behavior of 24Li-AC is ranked as follows: Freundlich > Langmuir > D-R. The superior fit of the Freundlich isotherm model suggests that the modified activated carbon surface is heterogeneous with a wide range of adsorption energies. This model's parameters, the Freundlich constant and exponent, reflect this heterogeneity and energy distribution. A high Freundlich constant indicates substantial adsorption capacity, while a low exponent implies a more linear adsorption isotherm^51^. In summary, the Freundlich isotherm model offers critical insights into CO_2_ adsorption on 24Li-AC, aiding in the optimization of their design and performance for CO_2_ capture applications.

Figure 15b depicts the adsorption isotherms for modified activated carbon (24Li-AC) at 30°C, demonstrating the relationship between adsorption capacity (q, in mmol/g) and pressure (P, in bar). This figure offers a detailed view of how the modified activated carbon performs under varying pressure conditions. The error bars in this figure represent the standard deviation from three independent experimental measurements, providing a visual representation of the data's variability and reliability. The error bars account for potential variations in temperature readings due to the accuracy of the thermometer used in the experiments. Similarly, the error bars consider potential inaccuracies in pressure readings resulting from the precision of the pressure gauge.

### Adsorption kinetic model correlation

Matching the experimental adsorption data to a set of conventional fixed models is a suitable technique for kinetic modeling due to the complexity of calculating kinetic factors and choosing the best model. Out of all the kinetic models listed in Table 8 that are used to describe the CO_2_ uptake process, the first and second models are the simplest in terms of explaining the kinetics of CO_2_ adsorption when compared to another models.

**Table 8:** Adsorption kinetic model.

Nonlinear form	Synthetic models
${q}_{t}={q}_{e}(1-{e}^{{k}_{1}t})$	First order
${q}_{t}=({q}_{e}^{2} {k}_{2}t)/( 1+{q}_{e}{k}_{2}t)$	Second order
${q}_{t}=\beta \text{ln}\left(\alpha \beta \right)+ \beta \text{ln}(t)$	Elovich equation
$q_{t} = q_{e} - \frac{{q_{e} }}{{\left( {1 + K_{2} .t} \right)}}$	Ritchie second order
${q}_{t}= {k}_{c}{t}^{0.5}$	Rate Controlling

The kinetic model results are presented in Table 9. The parameters for each model are listed separately by temperature from 30°C to 90°C. In physical adsorption, the first-order model is suitable for predicting the behavior of CO_2_ adsorption. The second-order model assumes that a reliable gas binding causes the interaction between adsorbent and adsorbate, which is more suitable for chemical adsorption when CO_2_ adsorption processes involve chemical interactions and chemical bonds.

**Table 9:** Parameters of kinetic models for LiOH-AC.

Kinetic model	Parameter	Temperature (K)			
		303.15	323.15	343.15	363.15
First order	q_e_	145.792	93.605	73.356	44.296
	K_1_	0.00589	0.0253	0.01394	0.02134
	R^2^	0.95995	0.96431	0.84882	0.8944
Second order	q_e_	153.900	95.423	77.021	45.736
	K_2_	0.00007	0.00052	0.00022	0.00065
	R^2^	0.99068	0.98615	0.92613	0.9428
Ritchie second order	q_e_	153.900	95.423	77.021	45.736
	k_2_	0.01077	0.0499	0.01732	0.02957
	R^2^	0.99068	0.98615	0.92613	0.9428
Elovich	α	0.188	250,453.093	1.597	94.931
	β	16.755	4.405	7.460	3.384
	R^2^	0.95476	0.96736	0.95798	0.94842
Rate controlling	k_id_	3.209	2.089	1.655	0.997
	R^2^	0.79462	0.61256	0.83156	0.75490

**Figure 16:**
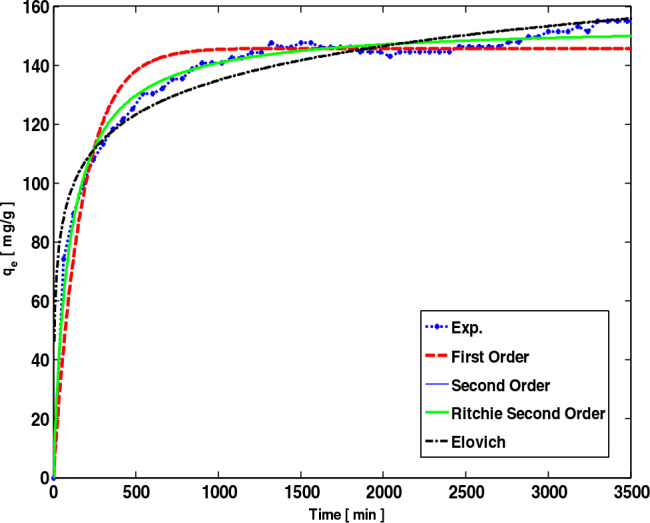
Kinetic models and experimental data for the kinetics modeling at 6 bar, 30 °C.

In Table 9, based on R^2^ values, the best model for the correlation at the temperature of 30 °C is second-order and Ritch second-order. Because AC is modified by LiOH, and this modification is used to increase the rate and increase the adsorption capacity due to the chemical process. Therefore, the second-order model shows chemical interactions well in modified adsorbent. In modified adsorbents, with a temperature rise of 70 °C and 90 °C, the model is suitable for displaying the kinetic state of the Elovich equation (Table 9). The Elovich equation describes an adsorption process as a reactions group, including the release of the bulk phase, surface emission, and active catalytic levels. Also, Elovich considers effective chemical energy changes about the level of surface coating and the reduction of carbon dioxide chemical adsorption. Therefore, it is suggested that CO_2_ adsorption for LiOH-AC is attributed to both chemical and physical adsorption modes (Fig. 16). These observations agree with the theory that adsorption sites occupy higher levels of energy at first in adsorption systems, in decomposition chemistry before adsorption.

### Adsorption thermodynamic parameters

The thermodynamic parameters and the behavior of the adsorption process can be identified by accomplishment an adsorption process at different temperatures. In engineering and environmental processes, both energy and entropy change parameters must be calculated to determine what processes will occur spontaneously. Gibbs free energy change, ΔG^0^, is an essential criterion of self-sufficiency. If the value of ΔG^0^ is negative, the reactions are performed spontaneously at a single temperature. The Gibbs free energy change (ΔG^0^), the enthalpy change (ΔH^0^) and the entropy change (ΔS^0^) are calculated using following equations:

5 $$Ln\left({K}_{d}\right)=\left(\frac{\Delta S^\circ }{R}\right)-\left(\frac{\Delta H^\circ }{RT}\right)$$

6 $${K}_{d}=\left(\frac{{P}_{i}-{P}_{f}}{{P}_{f}}\right)\left(\frac{V}{W}\right)$$

7 $$\Delta G^\circ =\Delta H^\circ -T\Delta S^\circ$$

**Figure 17:**
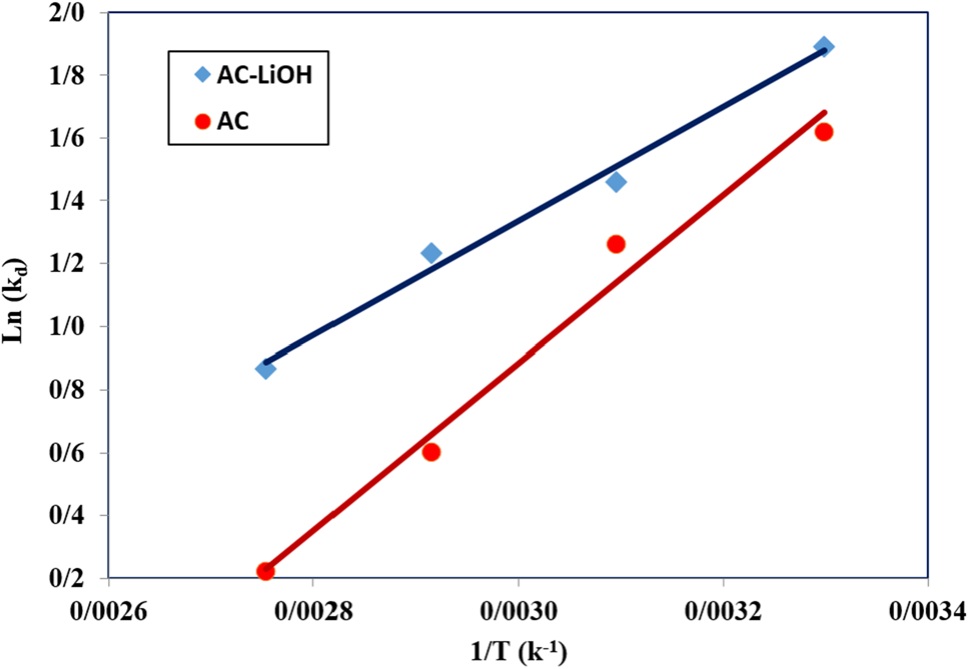
Ln k_d_ versus temperature before and after of LiOH modification.

By plotting of ln(K_d_) versus 1/T, the values of ΔH^0^ and ΔS^0^ are determined from the slope and intercept of the line, respectively. The parameters ΔG^0^, ΔH^0^, and ΔS^0^ are listed in Table 9 for AC before and after LiOH modification. The negative ΔS^0^ could be could be explained by the behavior of carbon dioxide molecules during the adsorption process. This is due to the randomness of the shape of the molecules arranged on the adsorbent surface. Besides, ΔH represents the type of CO_2_ adsorption process, whether in physical or chemical adsorption. ΔH^0^ in physical reactions is lower than 40 kJ/mol, while for chemical adsorption is 80 to 200 kJ/mol. Therefore, the calculated ΔH^0^ shows that the adsorption of natural CO_2_ is consistent with decreasing the amount of CO_2_ at high temperature (Fig. 17)^52^.

According to Table 10, the value of ΔH^0^ is positive, indicating that the adsorption reaction is endothermic. Also, the positive value of ΔG^0^ decreases with increasing the temperature, which indicates that the carbon dioxide adsorption process is desirable at 30 °C relative to 50 °C.

**Table 10:** Thermodynamic parameters of CO_2_ adsorption using AC before and after modification at 6 bar.

Adsorbent	ΔH° (kJ mol^−1^)	ΔS° (kJ mol^−1^ K^−1^)	ΔG° (kJ mol^−1^)			
			303.15 (K)	323.15 (K)	343.15 (K)	363.15 (K)
LiOH-AC	12.258	− 0.017	− 7.031	− 6.685	− 6.340	− 5.996
AC	16.118	− 0.031	− 6.618	− 5.991	− 5.364	− 4.738

### DFT simulation results

First, the modeling and simulation related to the structure of ACis discussed and the rate of adsorption of carbon dioxide by AC is presented based on the binding energy index. In the second part of the simulation, LiOH nano-models that perform the adsorption process to perfection are introduced, and then the binding energy related to the adsorption of carbon dioxide is reported in order to can be compared performance of the AC structures and LiOH nanoclusters. And finally, we will examine the performance of carbon dioxide adsorption by hybrid systems consisting of active carbon and LiOH structures.

### Structure of activated carbon

**Figure 18:**
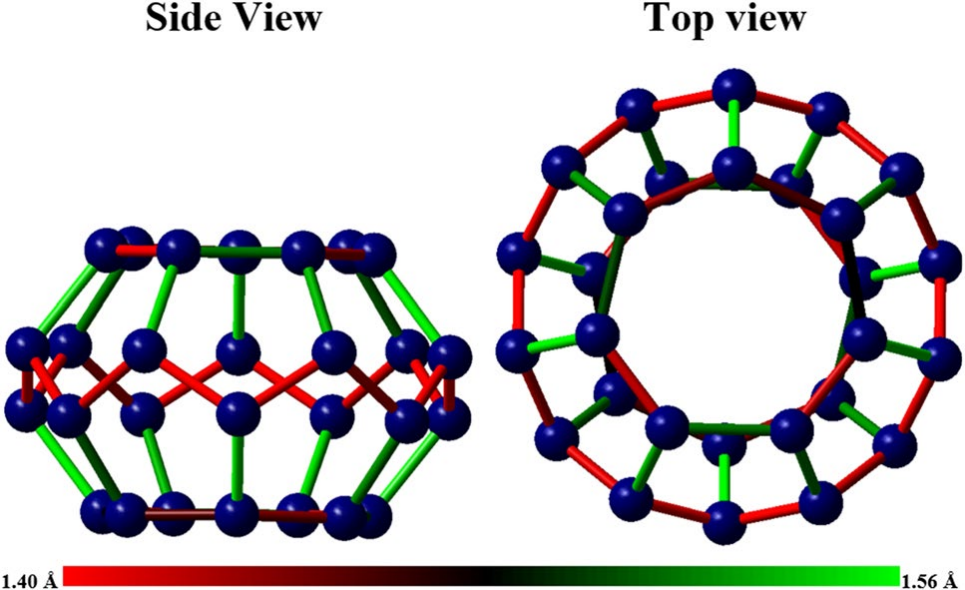
A modeled structure of AC with chemical formula C28, which fullerene includes pentagonal and heptagonal rings.

Experimental studies show that the active carbon structure can be assumed to be a fullerene composed of heptagonal and pentagonal rings^53^. Therefore, in this research, a fullerene with the same specifications has been designed and considered as a representative of the active carbon structure in modeling’s, whose geometry is shown in Fig. 18.

**Figure 19:**
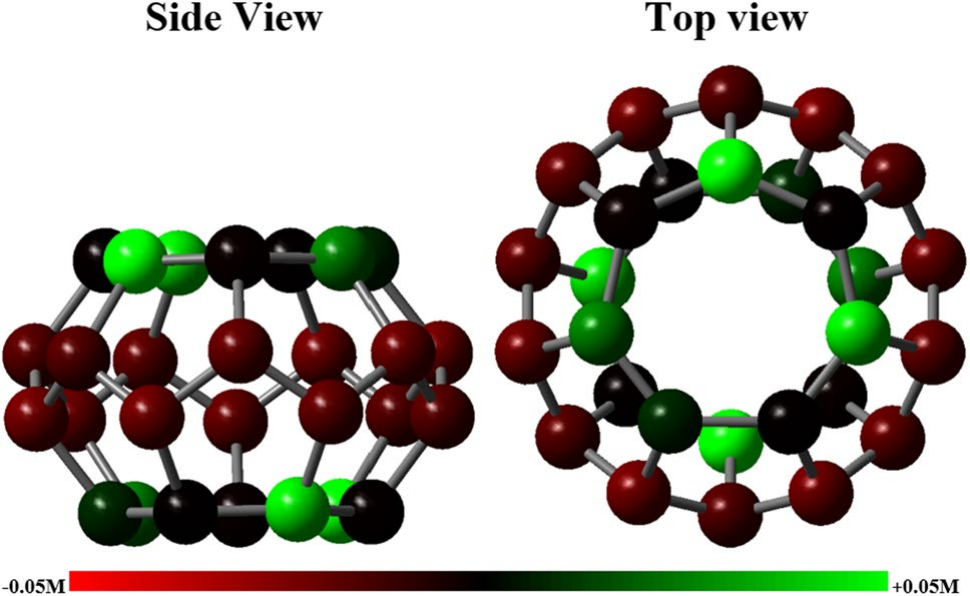
The electric charge distribution of the C28 structure on the Mulliken scale.

The above structure can be considered the smallest modeled structure for activated carbon, which consists of 28 carbon atoms and its largest diameter is about 5.33 angstroms. In this fullerene, the smallest bond is about 1.39 and the largest is about 1.56 angstroms. Figure 19 shows how the electrical charge distribution of the C_28_ structure is on the Mulliken scale^54^.

Based on the Mulliken charge distribution, it can be seen that the electronegativity of the atoms of the side ring of the structure is lower than that of the carbons at the top and bottom of the structure. Therefore, the probability of carbon dioxide adsorption from the top and bottom of the C_28_ structure is higher than from other sides. Now, using the binding energy index, the performance of carbon dioxide adsorption by AC is investigated^55^. The binding energy is obtained by the following relation:

8 $${E}_{b}={E}_{C\text{O}2}+{E}_{Structure}-{E}_{Combination}$$

Modeling based on DFT calculations shows that the structure of C_28_ in the optimal state adsorbs the carbon dioxide molecule to a distance of 3.3 angstroms and the corresponding binding energy is about 0.06 eV, from the side of the upper and lower face, that is, from the side of the heptagons of the structure. Of course, by applying pressure, the distance of carbon dioxide from the C_28_ absorbent structure decreases. It should be noted that the adsorption of carbon dioxide from the around of the C_28_ structure, near the pentagonal faces, is relatively weaker compared to its heptagonal faces. In the following, the adsorption of carbon dioxide by LiOH base structures will be investigated and finally, the issue of whether the presence of LiOH structures mixed with AC is effective in absorbing carbon dioxide.

### The role of LiOH nanostructures

**Figure 20:**
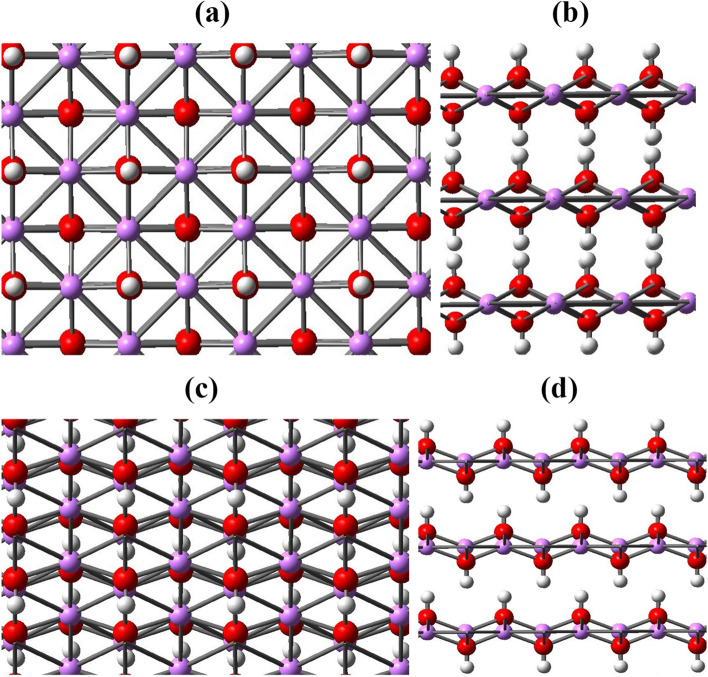
(**a**) A top view of the LiOH crystal structure, and (**b**) the corresponding side view of Figure (**a**). (**c**) An oblique view of the LiOH crystal structure, and (**d**) a side view of (**c**).

In this section, to investigate the role of LiOH in improving the performance of CO_2_ adsorption by AC, structures with dimensions comparable to the modeled AC structure were designed. Because if the LiOH crystal was considered, it would be necessary to use periodic boundary condition calculations, while a particle modelled for AC is a free-standing structure and Gaussian functions are used for high accuracy. Anyway, in this research, both due to computational considerations and considering the effects of nanoization that increases the surface interaction of materials, LiOH nanostructures are presented. In order to model a logical free-standing structure of the LiOH crystal structure, it is necessary to consider the LiOH salt structure from different aspects. The periodic structure of LiOH crystals from several directions is shown in Fig. 20.

**Figure 21:**
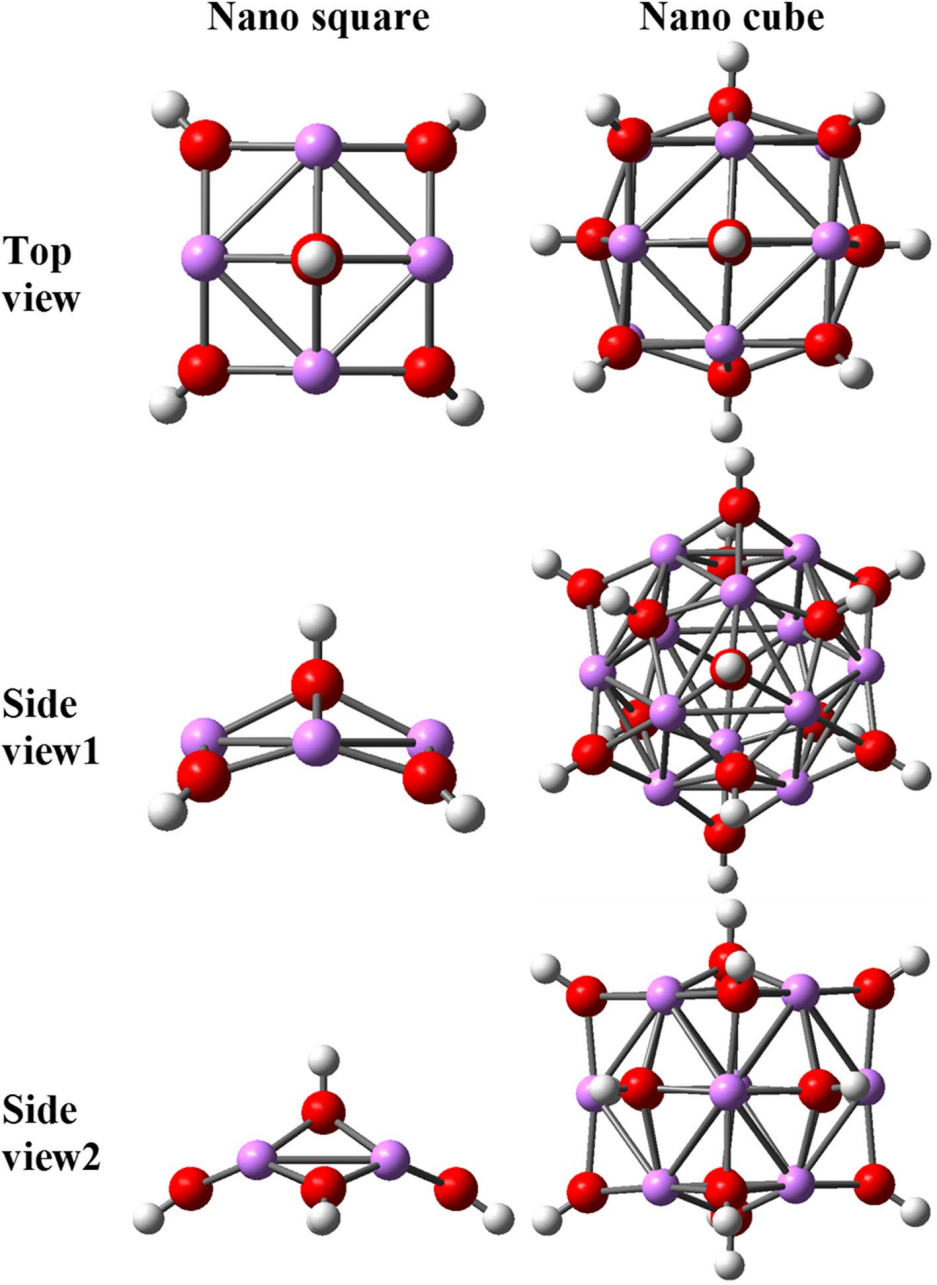
Two nano-square nanostructures with the formula Li_4_(OH)_5_ and nano-cube with the formula Li1_2_(OH)_14_ of LiOH in different views.

Considering the crystal structure of LiOH, it is clear that it has a layered structure. According to the monolayer structure of LiOH, two free-standing structures were designed to express the characteristics of the LiOH crystal. One of them is in the form of a nano-square, which is designed based on a repetitive pattern in the LiOH monolayer structure^56^; the other is a nano-cube, each face of which is similar to the repeating pattern in the LiOH crystal structure. Figure 21 shows the designed nano-square and nano-cube structure.

While on the around of the C_28_ structure, near the pentagonal faces, at a distance of 2.1 angstroms, the attraction force of carbon dioxide changes its place with repulsion, but nano square of LiOH at this distance optimally and stably with a binding energy of 0.11 eV absorb carbon dioxide, even this amount of binding energy appears in the nano cube at a distance of 2.4 angstroms.

The superiority of the nano-cube to the nano-square is its high symmetry, which is close to isohedral, as a result of which its performance is not dependent on the direction and acts isotropically. There is no significant difference in the binding energy between two lithium-based nanostructures. The binding energy values can be considered as the maximum value for the attraction of carbon dioxide by LiOH structures because of their small size.

Now, the adsorbent systems, which are composed of AC and LiOH, are investigated. For this work, the interaction of C_28_ fullerene and Li_4_(OH)_5_ nano-square structure was investigated simultaneously with carbon dioxide. In Table 11, the results of the calculations related to the composite absorbent system consisting of AC and LiOH are given for comparison with the pure absorbent systems of AC and LiOH. Table 11 shows the results of the calculations related to the composite adsorbent system consisting of AC and LiOH. To compare the results related to the binding energy of carbon dioxide with the modelled structure of the AC and nanostructures related to LiOH, it is presented separately.

**Table 11:** Binding energy along with the corresponding distance related to the adsorption of carbon dioxide by different adsorbent systems.

Adsorbent system and CO_2_	Binding energy (eV)	Distance of CO_2_ from the adsorber (Å)
C_28_ + Li_4_(OH)_5_ + CO_2_	0.13	2.4
C_28_ + CO_2_	0.06	3.3
Li_4_(OH)_5_ + CO_2_	0.11	2.1
Li_12_(OH)_14_ + CO_2_	0.11	2.4

According to the data in Table 10, it can be seen that with the addition of LiOH nanostructures along with activated carbon, the binding energy of carbon dioxide adsorption increases up to two times, and the corresponding distance decreases by about 1 angstrom. The noteworthy point is that the binding energy of hybrid systems for carbon dioxide adsorption is greater than the corresponding energy for the adsorption of LiOH nanostructures.

## Conclusion

The modification of activated carbon (AC) was successfully achieved using a 24% LiOH solution, significantly enhancing its CO_2_ adsorption capacity. The experimental design was conducted using the Central Composite Design (CCD) method, and the response surface methodology was employed to determine the adsorption capacity (mg/g) of the modified adsorbent. Key variables considered were temperature (°C), pressure (bar), LiOH concentration (mol/L), and adsorbent weight (g). The optimal conditions for maximizing CO_2_ adsorption capacity were found to be 30°C, 9 bar, 1 mol/L LiOH (24Li-AC), and 0.5 g of adsorbent. The results indicate that CO_2_ adsorption capacity increases with pressure and decreases with temperature. The incorporation of LiOH enhances the adsorbent properties by neutralizing the adsorbent surface. Quantitative analysis confirmed the experimental results using the Freundlich isotherm models. Adsorption kinetics analysis revealed that CO_2_ adsorption aligns with a second-order model at 30 °C and 50°C, and with the Elovich model at 70 °C and 90 °C. Thermodynamic studies indicated that CO_2_ adsorption by LiOH-AC is an endothermic process. In the computational aspect of this study, Density Functional Theory (DFT) simulations were performed to model and simulate the structure of AC and its CO_2_ adsorption behavior based on the binding energy index. LiOH nano-models were introduced to optimize the adsorption process, and the binding energy associated with CO_2_ adsorption was reported to compare the performance of AC structures and LiOH nanoclusters. Furthermore, the performance of hybrid systems composed of activated carbon and LiOH structures was evaluated. DFT results demonstrated that the hybrid systems exhibited superior CO_2_ adsorption capabilities, corroborating the experimental findings and highlighting the synergistic effects of combining AC with LiOH. These comprehensive findings provide a robust understanding of the modified AC's enhanced performance, validated through both experimental and theoretical approaches.

## Data Availability

The data used and analyzed during the current work is available from the corresponding author upon reasonable request.
